# Commentary: From the Editor's desk

**DOI:** 10.1155/S1463924680000028

**Published:** 1980


### Commentary

### A view from down under

After visits over the years to countries east of Suez, a lengthy sojourn in the capital cities of Australia affords an opportunity to review the situation with regard to laboratory automation in the geographical area usually referred to as the Far East.

Many of the countries included are unique and together they represent a wide range of economic, social and industrial developments, therefore generalised comment is not possible except in-so-far as the major suppliers of automatic instruments are based mainly in the 'west', and the features common to distant markets are apparent.

Sales and maintenance are carried out to a much greater extent by agents, long delays can occur before new instruments and developments are introduced and since many are not economically viable because of the length of supply lines or the small size of markets, the range of instruments available is considerably reduced. It is evident however, that when a manufacturer makes a determined effort to introduce equipment he can achieve remarkable success if only because of the lack of determined competition in many areas. The market is far from being uncritical however; some of the best evaluations of automatic instruments for clinical chemistry have been completed, for example, in Adelaide.

In developing countries such as Australia, the population to be served for health care might be comparatively small (14 million) but quality and service is high. The national economy is buoyant and in the recent past there has been considerable investment in the hospital sector. Some of the world's newest and best equipped hospitals are to be found in the major cities.

At the opposite end of the population scale, China, with approximately one quarter of the world's population (some 900 million) presents a somewhat different picture. Until recently there was little scientific contact outside the country, but the progress of science has been impressive. Chairman Mao's policy of not trailing behind the rest of the world but developing independently, together with equal care and opportunity for all has meant that the import of 'Western' technology has been minimal, the need for automation not being great because of the plentiful supply of labour and absence of centralisation. The overall picture in China, therefore, is of a wide distribution for instance of health care, with a very large number of relatively simple manual colorimeters and few centres in the large cities needing and using modest systems of automation. With the country's rapid economic advance and new outward look there is enormous development potential which in the more industrial areas of chemistry will probably be on conventional lines, but in the health sector, growth will almost certainly follow the already successful approach with a wide distribution of relatively small scale, broad-based facilities.

The usage and range of instruments in Japan differs little from those in other highly developed nations. A somewhat belated but now rapidly growing local interest in the design and production of automatic systems resulted in a number of Japanese made machines, some large and complex, being in use in the country but not as yet exported on any scale.

India also has a rapidly growing chemical instrument manufacturing industry, but it differs from the Japanese for two reasons. Firstly, in the absence of very large manufacturing firms interested and willing to enter the market, large specialist companies have had to develop, and since these do not have the advantage of already functioning efficient design engineering departments, the complexity of the machines which can be produced is restricted. Secondly, the requirements of the country are more for small, low cost, efficient, manually operated instruments rather than for large computer-controlled fast analysers. Since both China and India have similar requirements with matching and growing production, it is possible that by specialising in the type of instrument required by developing nations in general, they may find a useful outlet into Third World markets.

Nepal is an example of a small country which comparatively recently had no motor roads or vehicular access, let alone laboratory based medicine. Yet with WHO guidance and outside monetary aid, facilities are rapidly expanding outside Katmandu and like all other countries developing similarly, the requirement is for inexpensive, simply operated instruments which will function for many years without maintenance and independent of mains electricity supplies. Other countries in the area fall between the extremes mentioned, but as in the 'West' for those without advanced services and economies there are many instances where equipment designed for 'Western' civilisations has been installed at considerable expense, only to break down permanently at an early stage or at best, operate at a low level of efficiency. Occasionally however, there are to be found items of advanced technology such as mass spectrometers, operating at high efficiency under the care of dedicated and resourceful operators.

In summary, therefore, in the use of chemical automation, east of Suez things are different, but considering the very different conditions and the distances involved, the differences are not as great as might be expected.

*F.L. Mitchell*

### From the Editor's desk

As the *Journal of Automatic Chemistry* moves into its second volume I can report a steadily growing subscription list, a rapidly increasing interest from the commercial sector and a considerable input of papers for publication. Interest in the journal is now almost world wide and with the citation of many of our papers in other scientific journals the subscription should grow rapidly. Whilst there is often concern at the proliferation of new journals, the *Journal of Automatic Chemistry* attempts to be unique. It is multidisciplinary by the nature of its subject areas and also covers wider issues including education, management, and economics. Educational aspects have been widely covered in Volume I although no doubt there is still more to say. Management is the subject of Foreman's article in this issue and economic aspects are treated in some detail by Craig. The latter which was presented at clinical meetings at Brighton and Singapore, relates to a particular company's approach to costing, but is also of general applicability. Whilst these papers relate on the one hand to an industrial environment and the other to clinical laboratories each has value to readers in the other disciplines. Indeed it is the mechanism to promote the cross fertilisation of ideas and concepts between different disciplines that I see as one of the most important functions of the journal. Initially the concept was to foster the exchange of ideas between clinical and industrial users of automation but more recently the value of involving the discipline of process control was put to me.

### Automation and process control

I was recently invited to present a paper at the FACSS meeting in Philadelphia. One symposium there related to process control, and two facts immediately emerged from this meeting.

The first came from a questioner who stated that he had been sent on a short course entitled 'Automation and Computerisation'. He was very surprised that in fact the only subject taught was computerisation. He asked if there was not some other more appropriate course, definition or use of automation. His problem is of course not unique since, as has already been stated many times, there is no accepted definition of the term automation. This journal will hopefully provide the accepted base to judge automation. The short course described by Dr Betteridge [1] clearly provides a lead both within the UK and the world. Hopefully the Summer School will become an accepted course offered annually at Swansea.

The second fact that emerged was the role of the analytical chemist in automatic online analysis and the need for this Journal to cover this vital role of automation in its editorial material. Far more capital is invested in online process instrumentation than laboratory instrumentation. It is therefore surprising that a large technological gap exists between process and laboratory technology. Hirchfield [2] stated that computerisation was only a partial explanation for this and suggested that the main reasons were the cost and reliability of online instrumentation. However, a number of techniques such as microwave absorption, modified nmr and mass spectroscopic techniques have approached a stage where their potential for online instrumentation is a reality. In the symposium Oliver [3] also examined the role of the analytical chemist in the development online control system. Such interaction discussed by these authors at the meeting suggests a lively input of views to add to the journal.

### Choice of instrumentation

Factors influencing the choice of analytical instrumentation for the clinical laboratory are the subject of several papers in this issue. The predominate need which is applicable whether or not the instrumentation is purchased or constructed inhouse, is to provide a prior specification of the analytical requirements. In order that the equipment meets this requirement, any analytical, operational or economic constraints should be complied with to the complete satisfaction of the user. What is suitable instrumentation for a hospital may not be completely appropriate for industrial uses. However the approach to selecting the instrumentation can be and often is fairly similar for both purposes.

### Future of the *Journal of Automatic Chemistry*

On completion of the first volume it is only right and proper that we consider where the journal is going. The input of unsolicited new papers suggests almost overwhelmingly that, whilst a quarterly publication is economically viable, a more frequent publication could be justified on a purely editorial basis. Papers, particularly evaluations could then be published in the timescale appropriate to their needs. Views expressed by the readership are extremely valuable. I have had many verbal comments on my travels but few letters commenting directly on the content and general style. If you have any comments to make to me or the readership at large, please do not hesitate to write – communication is the aim of this journal.

### New technology in instrumentation

At the FACSS meeting and more recently in the UK I was able to appraise the design of the recently introduced Hewlett Packard UV/visible spectrometer. The instrument represents a significant advance in instrumentation for this technique and utilizes microprocessor technology in a very meaningful manner. In addition it also uses improved diode array technology to provide a very flexible and valuable automation tool. The instrument is described briefly in the new product section of this issue. As a straight replacement for a simple spectrometer it can perhaps be considered expensive. In reality the flexibility and the value of the sophisticated software packages suggest that the complete package of the spectrometer, pointer/plotter and tape deck are good value for money. It is, perhaps, a slight paradox that the design concepts integrated into the instrument remove the need to mechanically scan the optical spectral range by using diode arrays but use a mechanical scan to interrogate five cell positions. The system will undoubtedly open up new market areas particularly for process control applications. Users must be educated to take a new look at an old technique and learn to use the power of the system wisely. The instruments software and the external control facilities can be exploited to advantage.

REFERENCES

[1] Betteridge, B., *Journal of Automatic Chemistry* 1979, 1, 249.
[2] Hirschfield, T., Federation of Analytical Chemistry and Spectroscopy Societies.1979, Meeting paper 45.
[3] Oliver, R.T. Federation of Analytical Chemistry and Spectroscopy Societies 1979 Meeting paper 47.

### Forthcoming articles in the Journal of Automatic Chemistry

Among the articles which we hope to publish in future issues of the Journal of Automatic Chemistry are:

A microprocessor controlled scanning polarograph for solution labile compounds

An automatic collector for density gradients

Particle counting immunoassay (PACIA IV) – its application to the determination of human placental lactogen

Automated systems for the clinical laboratory – the users needs

Automated systems for the clinical laboratory – a manufacturers view of users needs

The evaluation of the Nova 2 ionised calcium instrument

The flexible dialyzer membrane – a source of error in plasma acreatinine determination

Automated determination of fatty acids in soaps and detergents using an AutoAnalyzer system

A compact automated microprocessor based stopped flow analyser

Microprocessor based monchromator controller

Design consideration involved in the development of an automated hydride evolution system for measurement of monogram amounts of arsenic and selenium by atomic spectroscopy

